# Wnt signalling modulates transcribed-ultraconserved regions in hepatobiliary cancers

**DOI:** 10.1136/gutjnl-2016-312278

**Published:** 2016-09-12

**Authors:** Pietro Carotenuto, Matteo Fassan, Rosantony Pandolfo, Andrea Lampis, Caterina Vicentini, Luciano Cascione, Viola Paulus-Hock, Luke Boulter, Rachel Guest, Luca Quagliata, Jens Claus Hahne, Rachel Ridgway, Tam Jamieson, Dimitris Athineos, Angelo Veronese, Rosa Visone, Claudio Murgia, Giulia Ferrari, Vincenza Guzzardo, Thomas Ronald Jeffry Evans, Martin MacLeod, Gui Ji Feng, Trevor Dale, Massimo Negrini, Stuart J Forbes, Luigi Terracciano, Aldo Scarpa, Tushar Patel, Nicola Valeri, Paul Workman, Owen Sansom, Chiara Braconi

**Affiliations:** 1 The Institute of Cancer Research, London, UK; 2 Department of Medicine, University of Padua, Padua, Italy; 3 ARC-NET Research Centre, University of Verona, Verona, Italy; 4 Institute of Oncology Research, Bellinzona, Switzerland; 5 Institute of Cancer Sciences, University of Glasgow, Glasgow, UK; 6 MRC Human Genetics Unit, University of Edinburgh, Edinburgh, UK; 7 MRC Centre for Regenerative Medicine, University of Edinburgh, Edinburgh, UK; 8 Molecular Pathology Division, Institute of Pathology, University of Basel, Basel, Switzerland; 9 Cancer Research UK Beatson Institute for Cancer Research, Glasgow, UK; 10 Department of Medical, Oral and Biotechnological Sciences, G. d'Annunzio University, Chieti, Italy; 11 School of Biosciences, Cardiff University, Cardiff, UK; 12 University of Ferrara, Ferrara, Italy; 13 Mayo Clinic, Jacksonville, Florida, USA; 14 The Royal Marsden NHS Foundation Trust, London and Surrey, UK

**Keywords:** HEPATOCELLULAR CARCINOMA, CHOLANGIOCARCINOMA, BILARY DUCT CARCINOMA, RNA EXPRESSION

## Abstract

**Objective:**

Transcribed-ultraconserved regions (T-UCR) are long non-coding RNAs which are conserved across species and are involved in carcinogenesis. We studied T-UCRs downstream of the Wnt/β-catenin pathway in liver cancer.

**Design:**

Hypomorphic Apc mice (*Apcfl/fl*) and thiocetamide (TAA)-treated rats developed Wnt/β-catenin dependent hepatocarcinoma (HCC) and cholangiocarcinoma (CCA), respectively. T-UCR expression was assessed by microarray, real-time PCR and in situ hybridisation.

**Results:**

Overexpression of the T-UCR uc.158− could differentiate Wnt/β-catenin dependent HCC from normal liver and from β-catenin negative diethylnitrosamine (DEN)-induced HCC. uc.158− was overexpressed in human HepG2 versus Huh7 cells in line with activation of the Wnt pathway. In vitro modulation of β-catenin altered uc.158− expression in human malignant hepatocytes. uc.158− expression was increased in *CTNNB1*-mutated human HCCs compared with non-mutated human HCCs, and in human HCC with nuclear localisation of β-catenin. uc.158− was increased in TAA rat CCA and reduced after treatment with Wnt/β-catenin inhibitors. uc.158− expression was negative in human normal liver and biliary epithelia, while it was increased in human CCA in two different cohorts. Locked nucleic acid-mediated inhibition of uc.158− reduced anchorage cell growth, 3D-spheroid formation and spheroid-based cell migration, and increased apoptosis in HepG2 and SW1 cells. miR-193b was predicted to have binding sites within the uc.158− sequence. Modulation of uc.158− changed miR-193b expression in human malignant hepatocytes. Co-transfection of uc.158− inhibitor and anti-miR-193b rescued the effect of uc.158− inhibition on cell viability.

**Conclusions:**

We showed that uc.158− is activated by the Wnt pathway in liver cancers and drives their growth. Thus, it may represent a promising target for the development of novel therapeutics.

**Summary box**

**What is already known on this subject?**

- The Wnt/β-catenin pathway is frequently activated in liver cancer. The understanding of the molecular mechanisms of Wnt deregulation is necessary to develop more specific and effective therapeutics.
- Transcribed-ultraconserved regions (T-UCRs) are long non-coding RNAs that harbour ultraconserved elements and are altered in human carcinomas.

**What are the new findings?**

- We demonstrate that T-UCRs are downstream mediators of the Wnt/β-catenin pathway in three different species, and we provide evidence of their role as competing endogeneous long non-coding RNAs.
- We have identified a mediator that is downstream of the Wnt/β-catenin pathway and specific for cancer development, improving the development of effective and selective novel therapeutics.

**How might it impact on clinical practice in the foreseeable future?**

- We have studied several cohorts of human liver cancers and observed that deregulation of uc.158− is shared between hepatocarcinoma and cholangiocarcinoma, providing interesting insights into the molecular biology of these diseases and the implication for clinical trial design.

## Background

Hepatocellular carcinoma (HCC) is the sixth most common malignancy in the world.^1^ Despite the progress of recent decades, the outcome of patients with advanced HCC remains poor, and mortality for liver cancer has increased worldwide.^1^ Sorafenib is the only drug which is effective in advanced HCC with an improvement of about 3 months in median overall survival.^2^ Novel therapies are urgently needed, but their development is challenged by the limited understanding of the molecular mechanisms underlying HCC progression and the subsequent lack of identified targets that drive liver transformation.

The Wnt pathway is frequently activated in HCC.^3^ ^4^ Various attempts have been made to develop therapeutics targeting the Wnt pathway components, and some of these are now under clinical evaluation.^5^ However, the challenges faced in the development of Wnt inhibitors, such as specificity and toxicity, demand a better understanding of the molecular mechanisms involved in Wnt deregulation, in order to inform the design of more specific and more effective compounds. The canonical Wnt pathway (or Wnt/β-catenin pathway) leads to an accumulation of β-catenin in the cytoplasm that can be translocated into the nucleus and activate transcription factors.^3^ A recent analysis of human HCC has shown that the Wnt pathway is activated in 49% of HCC and that almost half of these had features consistent with the activation of the Wnt/β-catenin pathway.^6^ These data are in line with an integrated analysis of somatic mutations which revealed activating mutations of β-catenin (*CTNNB1*) in 33% of human HCC. In addition, mutations of *CTNNB1* are mutually exclusive with mutations in *AXIN1*.^7^

Non-coding RNAs (ncRNA) are implicated in liver carcinogenesis.^8–10^ We and others have provided data to support the involvement of small ncRNAs, such as microRNAs, in the modulation of liver cell proliferation and apoptosis.^10–13^ Despite several studies linking ncRNA and the Wnt pathway, most of them focused on the modulation of Wnt signalling proteins by microRNAs, little is known about the effect of this pathway on ncRNA.^14–17^ Increasing evidence points to an important regulatory role of long ncRNAs in cellular processes and disease phenotypes. Sequence conservation across species has been postulated to indicate that a given ncRNA may have a cellular function. A genome-wide survey identified 481 genomic sequences with a size >200 bp that showed a remarkable conservation with 100% identity across human, mouse and rat genomes.^18^ These ultraconserved regions (UCR) were shown to be transcribed as ultraconserved ncRNAs (T-UCR). They exhibit distinct profiles in various human cancers and modulate cellular proliferation and apoptosis.^19–25^ We have previously shown that T-UCRs are aberrantly expressed in HCC and have a role in the malignant growth of human and murine hepatocytes.^25^ In this study, we aim to investigate the effect of Wnt/β-catenin signalling on T-UCRs in order to find downstream effectors of this pathway that can be targeted for the development of novel and more specific therapeutics.

## Methods

### Animal models

All animals were handled in strict accordance with good animal practice as defined by the relevant national and/or local animal welfare bodies, and all animal works were approved by the appropriate committees. Experiments were carried out in compliance with UK Home Office Animal (Scientific Procedures) Act 1986 and the EU Directive 2010. Hypomorphic mutant (*Apc*580s/580s) *Apcfl/fl* mice were used and compared with *Apc+/+* mice. Model generation and demonstration of the activation of Wnt/β-catenin pathway in this HCC model have already been reported.^26^ We extracted RNA from the liver of *Apcfl/fl* (tumour with activation of Wnt/β-catenin) and *Apc*+/+ (normal liver). *TAA rat tumour model* was used as a model of rat cholangiocarcinoma (CCA). Generation of this model and activation of Wnt pathway in this tumour model have already been reported.^27^ ICG-001 (5 mg/kg) (Tocris, Bristol, UK) or C-59 (20 mg/kg) (Cellagen Technologies, San Diego, California, USA) was given by intraperitoneal injection. The vehicle for this was physiological saline (Sigma-Aldrich, Sigma, St Louis, Missouri, USA). Control animals were given vehicle alone. In all cases, inhibitors and vehicle were given three times per week.^27^ We extracted RNA from the liver of TAA rat (tumour) or saline control (normal liver), as well as from TAA rats treated with ICG-001, C59 or saline control. *Axin1fl/fl/Cre* mouse model was used to investigate the effect of *Axin1* disruption in the hepatocytes. This model was generated by crossing mice homozygous for the *Axin1fl/fl* allele with *AhCre* mice, as previously reported.^28^ We extracted RNA from the liver of *Axin1fl/fl/Cre* and *Axin1+/+/Cre* mice. *Diethylnitrosamine-induced HCC*: C57BL/6, 14 days old male mice were intraperitoneally injected with diethylnitrosamine (DEN) (40 μL of a solution containing 10 mg/mL DEN in PBS:80 mg/kg). Mice were sacrificed at 36 weeks after DEN injection; liver was assessed for macroscopic tumour number and size, and normal liver and tumour samples were placed into RNA later, snap-frozen in liquid nitrogen or fixed in neutral buffered formalin overnight at 4°C for the assessment of β-catenin.

### Rapid Apc inactivation in *Apcfl/fl* mice

To investigate the phenotype of conditional deletion of Apc, *AhCre+Apcfl/fl* and *AhCre+Apc+/+* progeny were used as previously described.^29^ Mice were given daily intraperitoneal injections of β-napthoflavone (80 mg/kg) as previously reported.^30^ After 4 days, mice were killed and liver removed, weighed and placed into RNA later (Sigma, St Louis, Missouri, USA) or fixed in formalin. For BrdU labelling, mice were injected with 0.25 mL of BrdU (GE Healthcare, Amersham, UK), 2 hours prior to sampling, and staining was performed using an anti-BrdU antibody conjugate (#347580, BD Biosciences, San Jose, California, USA; 1:150). Staining for mouse β-catenin was performed on sections from tissue samples fixed at 4°C for <24 hours in 10% formalin prior to processing using the antibody (#610154, BD Biosciences; 1:50).

### T-UCR expression profiling in mice

RNA from the liver of *Apcfl/fl* (n:3), *Apc+/+* (n:3) and DEN-treated mice (n:3) were used. mRNA was purified from total RNA after removal of rRNA (mRNA-ONLY Eukaryotic mRNA Isolation Kit, Epicentre, Madison, Wisconsin, USA). Then, each sample was amplified and transcribed into fluorescent cRNA along the entire length of the transcripts without 3′ bias, using a random priming method. The labelled cRNAs were hybridised onto the mouse LncRNA Array V.2.0 (Arraystar, Rockville, Maryland, USA), which also included probes for protein-coding RNAs. The arrays were scanned by the Agilent Scanner G2505C (Agilent Technologies, Santa Clara, California, USA). Agilent Feature Extraction software (V.11.0.1.1) was used to analyse the acquired array images. Quantile normalisation and subsequent data processing were performed using the GeneSpring GX V.11.5.1 software package (Agilent Technologies). After quantile normalisation of the raw data, differentially expressed LncRNAs with statistical significance between groups (*Apcfl/fl* vs *Apc+/+*; *Apcfl/fl* vs DEN-HCC) were identified through volcano plot filtering, with a threshold fold change ≥2.0 and p≤0.05.

### Human tissues

The human CCA tissues were collected under approval of the Ethical Committee for Clinical Research of Padua and the University of Edinburgh ethics committee. Healthy liver was provided by the NHS Scotland Research (NRS) BioResource, NHS Lothian. All tissues were collected with informed consent. HCC tissues were provided by the University Hospital Basel and University of Ferrara after appropriate ethical approval. For the CCA tissue microarray (TMA), a retrospective series (1990–2011) of 102 surgically resected primary biliary cancers, including 85 CCA and 17 gall bladder carcinoma specimens, were retrieved from the formalin-fixed paraffin-embedded (FFPE) archives of the Department of Pathology and Diagnostics and the ARC-NET biobank of the University and Hospital Trust of Verona after appropriate ethical approval. CCAs were classified according to WHO 2010 as intrahepatic cholangiocarcinoma (ICC, n=54) and extrahepatic cholangiocarcinoma (ECC; n=31). Five 1 mm core TMAs were assembled. Additional 14 non-neoplastic controls were included in the TMAs. Three tissue cores per case were analysed.

### Statistical analysis

Statistical analyses were performed by GraphPad Prism 6 (La Jolla, California, USA). Results are expressed as mean±SD, unless indicated otherwise. Groups that were normally distributed were compared with either a two-tailed Student's t-test (for analysis of two groups) or using one-way analysis of variance to compare multiple groups. Non-parametric data were analysed using a Wilcoxon-Mann-Whitney U test when comparing two groups. Significance was accepted when p<0.05. *p:0.05–0.01; **p:0.009–0.001; ***p<0.001.

Additional methods can be found in the online supplementary information, along with the primer sequences (see online supplementary table S1).

10.1136/gutjnl-2016-312278.supp1 supplementary data

## Results

### T-UCR profiling in HCC mouse models

**Figure 1:**
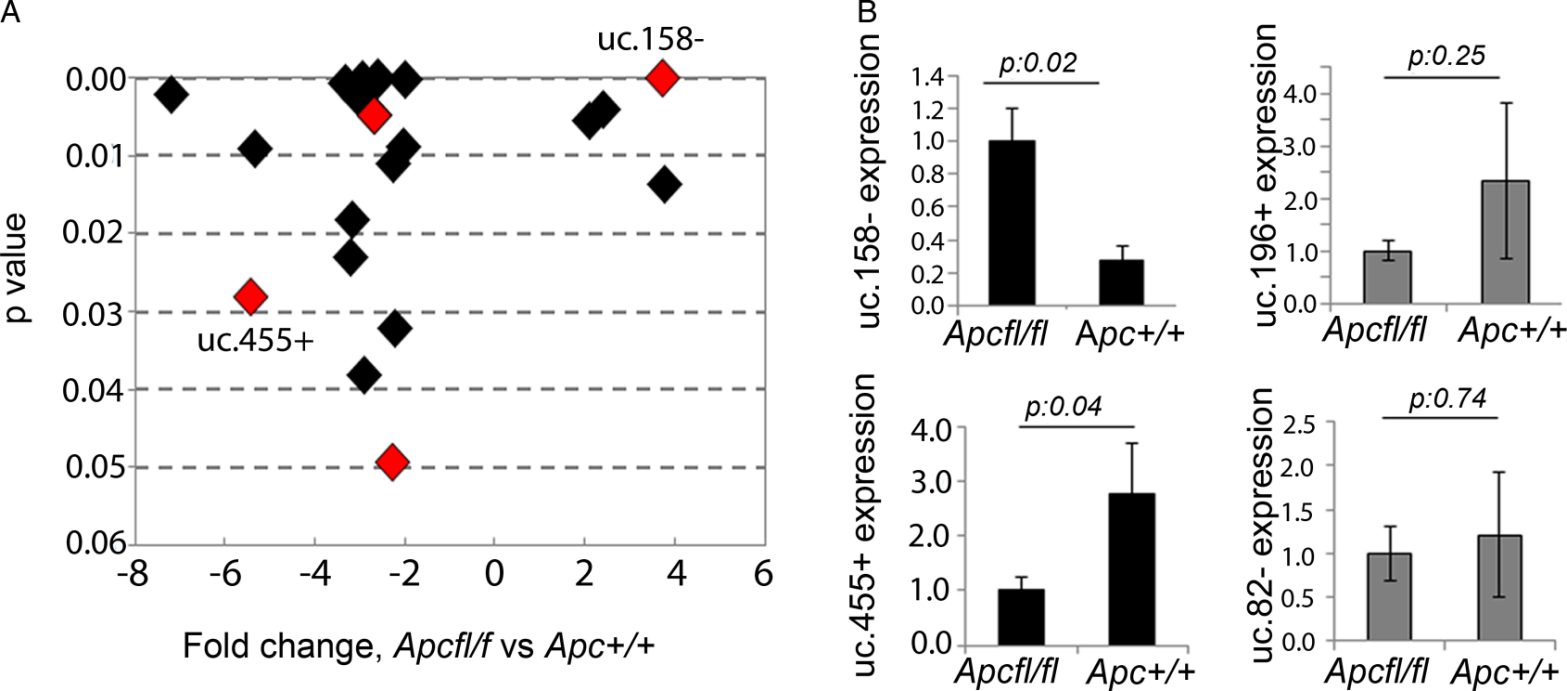
Transcribed-ultraconserved regions (T-UCR) profiling in an *Apc* hypomorphic hepatocarcinoma (HCC) mouse model. (A) T-UCRs aberrantly expressed in *Apcfl/fl* (n:3) versus control *Apc+/+* (n:3) mice livers. Fold change has been plotted against p values, with lowest p values being on the top of the y-axis. T-UCRs highlighted in red are those that were common across the two profiling comparing *Apcfl/fl* versus *Apc+/+* and comparing *Apcfl/fl* versus diethylnitrosamine (DEN)-induced HCC (n:3). (B) Validation of T-UCR expression by real-time PCR. Bars represent mean±SD of four samples.

Hypomorphic Apc mice (*Apcfl/fl*) were previously shown to develop HCC with activation of the Wnt/β-catenin pathway (nuclear localisation of β-catenin and activation of Wnt-related genes).^26^ To assess T-UCRs downstream of the Wnt pathway, we profiled ncRNA expression by microarray in the liver of *Apcfl/fl* and control *Apc+/+* mice. Twenty-two T-UCRs were aberrantly expressed (figure 1A, see online supplementary table S2), with four upregulated and eighteen downregulated, greater than twofold, in tumour tissues from *APCfl/fl* compared with normal liver from *Apc+/+*. To verify if these T-UCRs were specifically altered by Wnt/β-catenin or just affected by the malignant transformation, we profiled T-UCR expression in the livers of mice treated with DEN, which developed a median of 17 (range: 13–36) tumours per liver with no nuclear activation of β-catenin (see online supplementary figure S1). Interestingly, we found that four T-UCRs could further differentiate *Apcfl/fl*-HCC from DEN-induced HCC (figure 1A, see online supplementary table S3). Expression of uc.158−, uc.455+, uc.196+ and uc.82− was assessed by real-time PCR in the liver tissues of a second series of *Apcfl/fl* and control *Apc+/+* mice (figure 1B). uc.158− and uc.455+ were confirmed to be significantly upregulated and downregulated, respectively, in Wnt-dependent HCC. uc.158− is an intergenic 224 nt long UCR located on chromosome 13 in the mouse and on chromosome 5 in the human genome. uc.455+ is a 245 nt long UCR located on chromosome 2 in the mouse and on chromosome 20 in the human genome, where it overlaps in antisense the RNA-binding protein motif 39 (*RBPM39*) gene. Given the fact that uc.158− does not overlap protein-coding genes, and that intergenic lncRNAs have been shown to be more stable,^31^ we focused on uc.158− for further studies.

### Uc.158− expression is downstream of Wnt signalling in human liver cancer cells

**Figure 2:**
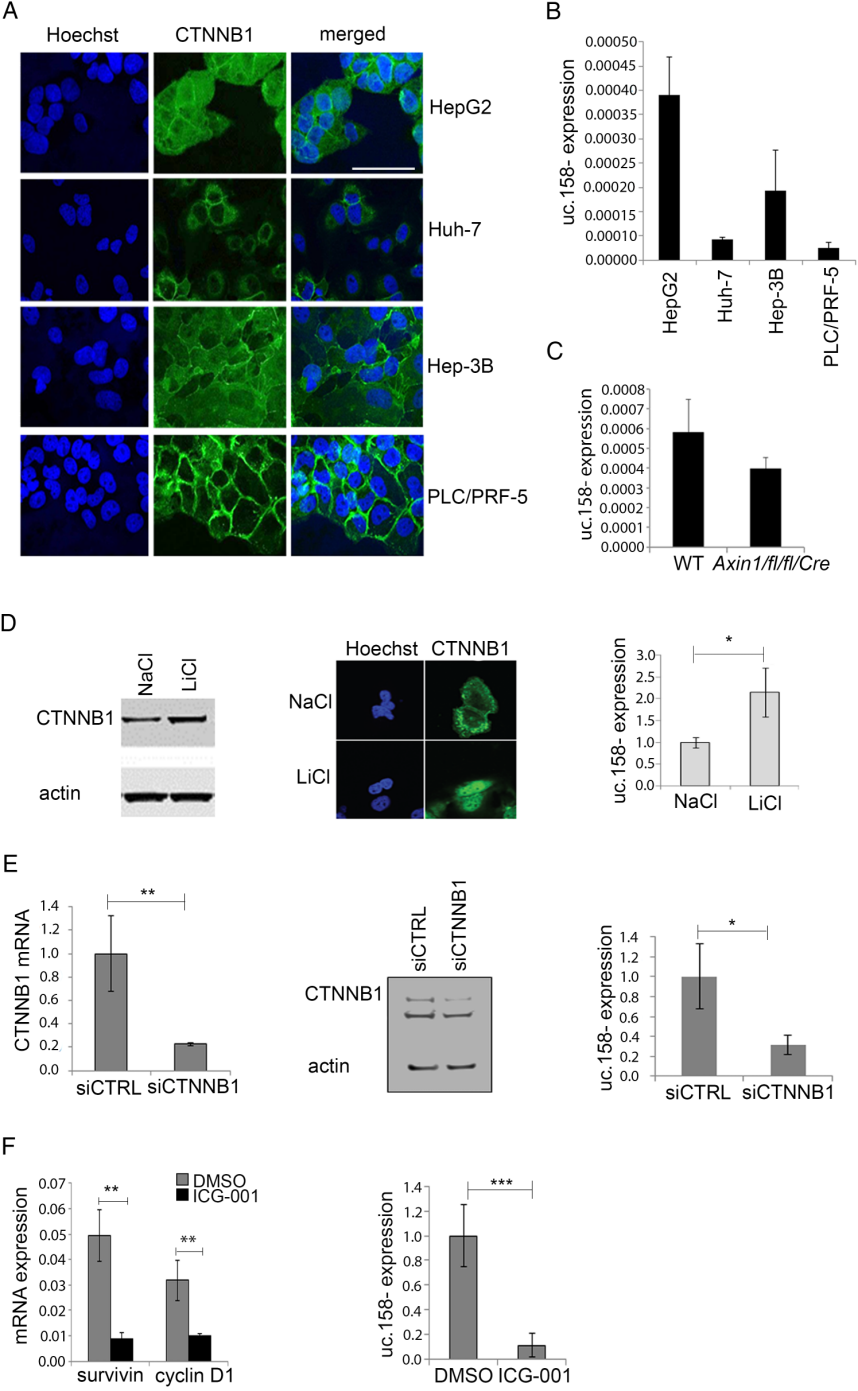
uc.158− expression is dependent on Wnt/β-catenin pathway in human malignant hepatocytes. (A) Immunofluorescence was performed for β-catenin (CTNNB1) and Hoechst in a panel of malignant hepatocytes. Scale bar: 50 μm (B) Expression of uc.158− was assessed in the same panel of malignant hepatocytes by real-time PCR. Bars represent mean±SD of three independent samples. (C) uc.158− expression was assessed by real-time PCR in the liver of wild-type (WT) and *Axin1fl/fl/Cre* mice. Bars represent mean±SD of six samples. p>0.05. (D) Huh-7 cells were treated with 25 mM LiCl or diluent control for 24 hours and cells collected to assess induction of β-catenin protein expression by western blotting, induction of nuclear localisation by immunofluorescence and expression of uc.158− by real-time PCR. Bars represent mean±SD of three experiments. (E) HepG2 cells were transfected with siRNA against *CTNNB1* (siCTNNB1) or siRNA CTRL (50 nM) for 48 hours, and cells collected to assess reduction in expression of CTNNB1 mRNA, protein expression and expression of uc.158− by real-time PCR. Bars represent mean±SD of three experiments. (F) HepG2 cells were treated with 50 μM ICG-001 for 24 hours, and cells collected to assess downregulation of drug-target mRNAs and expression of uc.158− by real-time PCR. Bars represent mean±SD of three experiments.

To understand the human relevance of previous findings, we assessed the expression of uc.158− in human malignant hepatocytes. HepG2 cells are known to harbour mutations in exon 3 of the β-catenin gene (*CTNNB1*), while Huh-7 cells are known to be wild type (WT) for *CTNNB1* and other genes of the Wnt pathway.^6^ The Wnt pathway is activated in HepG2 cell lines, but not in Huh-7, in line with the mutational status of these cells (see online supplementary table S4 and figure S2A, B). Global lncRNA expression analysis revealed that uc.158− is increased in malignant hepatocytes in comparison with normal human hepatocytes and that overexpression is greater in HepG2 than in Huh-7 (see online supplementary figure S2C). We then extended our studies to a panel of malignant hepatocytes. Immunofluorescence showed that β-catenin localises in the nuclei of HepG2 cells and Hep-3B, although to a lesser extent (figure 2A), as previously described.^32^ Interestingly, the expression of uc.158− reflects this finding, with the highest expression being in HepG2 (figure 2B). PLC/PRF-5 cells had a very low expression of uc.158− and did not show localisation of β-catenin despite their mutation in *AXIN1* 33 (figure 2A, B). To further study this finding, we analysed the expression of uc.158− in the liver of mice genetically engineered to harbour deletion of *Axin1*. These animals (*Axin1fl/fl/Cre*) were shown to develop microscopic foci of HCC in the absence of nuclear localisation of β-catenin.^28^ In line with previous findings, we did not detect any change in the expression of uc.158− in the liver of *Axin1fl/fl/Cre* in comparison with WT mice (figure 2C).

To further validate the dependency of uc.158− on the Wnt/β-catenin pathway, we assessed its expression after modulation of β-catenin in vitro. Treatment of Huh-7 cells with LiCl induced activation of β-catenin and an increase in the expression of uc.158− (figure 2D). Inhibition of β-catenin by siRNA or by the Wnt/β-catenin inhibitor ICG-001 induced a decrease in the expression of uc.158− in HepG2 cells (figure 2E, F).

### Uc.158− expression in human HCC tissues

**Figure 3:**
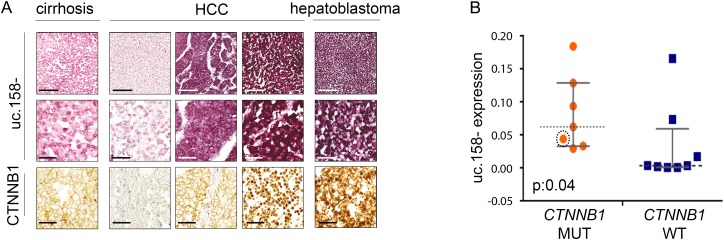
uc.158− expression in human hepatocarcinoma (HCC) tissues. (A) Formalin-fixed paraffin-embedded human liver tissues have been analysed for the expression of uc.158− by RNA in situ hybridisation and for CTNNB1 protein expression by immunohistochemistry. Scale bars: 200 μM for the top and bottom line, 50 μM for the second line. (B) DNA and RNA were extracted from 15 frozen HCC tissues and used for uc.158− expression by real-time PCR and sequencing analysis for *APC, AXIN1* and exon 3 of *CTNNB1*. The circled orange dot represents a case with mutation of *APC*. Bars represent median with IQR. Mann-Whitney test, exact two-tailed p value: 0.04.

**Figure 4:**
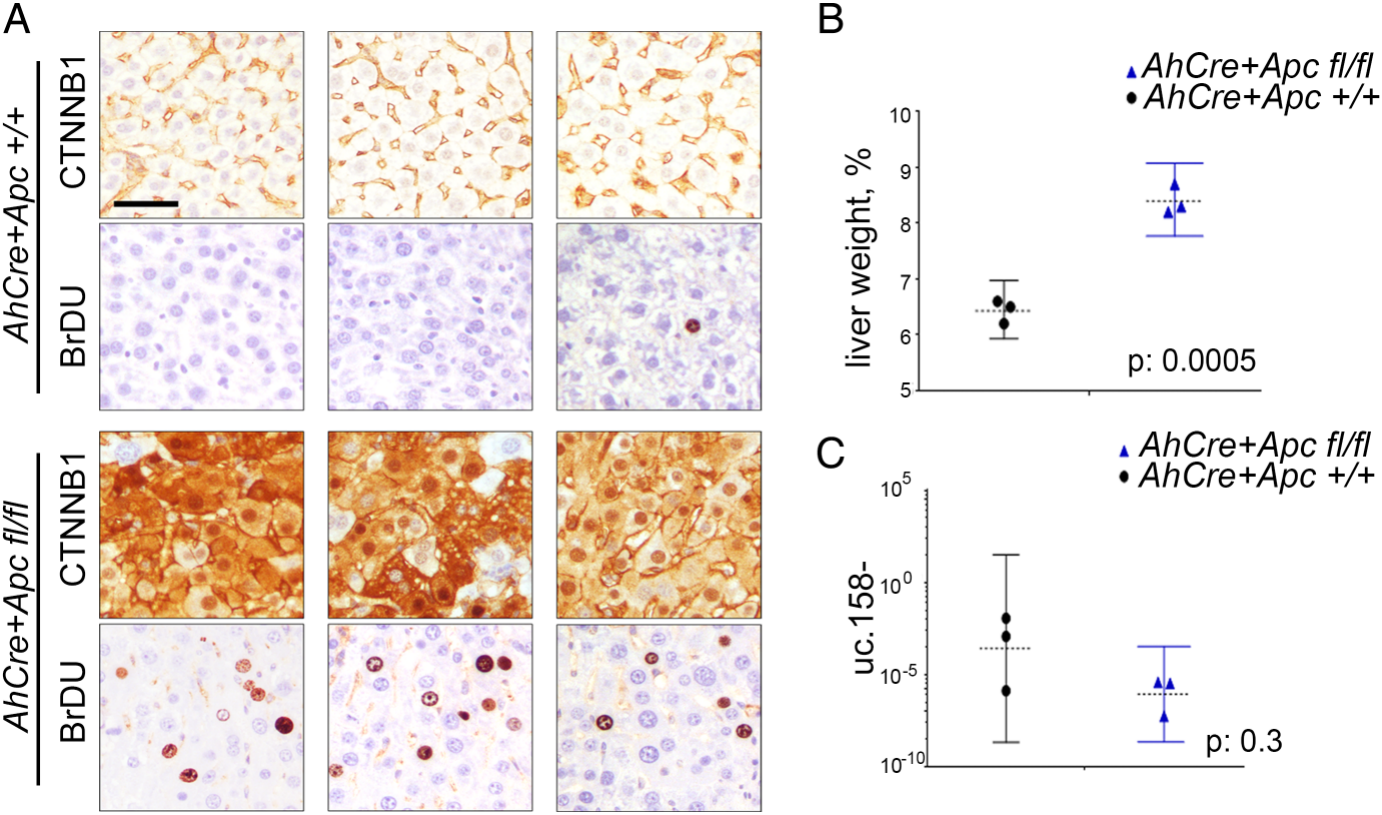
*Apc* deletion does not induce uc.158− in the absence of tumour. Conditional *Apc* deletion induced by β-napthoflavone injection in *AhCre+Apc+/+* and *AhCre+Apcfl/fl* mice on day 1. Mice (n=3 for each group) were euthanised on day 4, and liver weighed and processed for downstream analyses. (A) IHC analysis for CTNNB1 and BrDU. Scale bar: 10 μM (B) Liver weight is expressed as percentage of the whole body weight (median and range). (C) Expression of uc.158− (median and range).

We have previously shown that T-UCRs are transcribed as independent transcripts which are longer than the UCRs described by Bejerano.^25^ To characterise the 5′-end of the transcript including uc.158−, we performed a rapid amplification of cDNA ends (RACE). We have shown that uc.158− is transcribed in antisense and that the 5-end is characterised by additional 306 nucleotides upstream of the known uc.158− (see online supplementary figure S3). To confirm the transcript is transcribed in antisense, we performed in situ hybridisation in FFPE human liver tissues. We used three different probes: S1 which recognised the sense uc.158+ and AS1 and AS3 which both could detect the antisense uc.158−. In line with our RACE experiments, S1 did not show any signal, confirming that uc.158− is transcribed in antisense (see online supplementary figure S3). We next analysed a series of human FFPE liver tissues. Cirrhotic tissues were negative for uc.158− expression, while HCC tissues were positive. However, it is of note that HCC with negative or membranous expression of β-catenin by immunohistochemistry (IHC) had a weak/moderate cytoplasmic expression, while liver tumours with nuclear β-catenin showed the most intense signal (figure 3A, see online supplementary table S5). Next, we studied a second cohort of human frozen HCCs where *CTNNB1*, *APC* and *AXIN1* genes were analysed by sequencing (as previously described^34^). The median expression of uc.158− was significantly higher in the mutated group (which included six *CTNNB1*-mutated and one *APC*-mutated HCC) than in WT controls (figure 3B). However, isolated WT HCCs showed an increased expression of uc.158−, suggesting that in rare cases the source of uc.158− may be alternative to the mutations studied. Indeed, hepatitis liver tissues showed negative non-neoplastic hepatocytes and positive lymphocytes (see online supplementary figure S4A), suggesting that the stroma may represent a source of uc.158−. To understand if uc.158− is specific to malignant cells or can be expressed also in non-tumour hepatocytes, we looked at uc.158− expression in the liver of a mouse model with conditional *Apc* deletion (*AhCre+Apcfl/fl*), which presents an increase in liver weight and proliferation, but not tumours. Indeed, we noticed that uc.158− expression was not changed following activation of the Wnt/β-catenin pathway in absence of cancer (figure 4).

### Uc.158− in CCA

**Figure 5:**
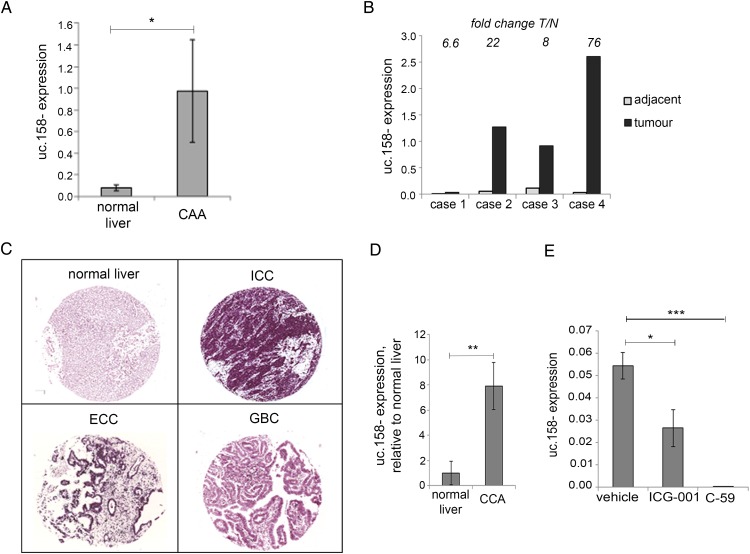
uc.158− expression in cholangiocarcinoma. (A) RNA was extracted from frozen intrahepatic cholangiocarcinoma (CCA) (n: 5) and normal liver (n:8) tissues and assessed for uc.158− expression by real-time PCR. Bars represent mean±SE. (B) In four cases, CCA and matched adjacent tissues from the same patients were available. Bars represent uc.158− expression in tumour tissue relative to the expression in the adjacent normal tissue. Fold change tumour/normal (T/N) is reported for each case on the top of the bars. (C) A tissue microarray of formalin-fixed paraffin-embedded human tissues including 54 intrahepatic cholangiocarcinoma (ICC), 31 extrahepatic cholangiocarcinoma (ECC), 17 gallbladder cancers (GBC) and 8 normal liver tissues was assessed for expression of uc.158− by RNA in situ hybridisation (ISH) with AS3 probe. Pictures of representative cases are shown. (D) RNA was extracted from the liver of thiocetamide (TAA)-treated (with CCA) or vehicle-treated (normal) rats for 26 weeks and analysed for uc.158− expression by real-time PCR. Bars represent mean±SD of three samples. (E) Rats with TAA-induced CCA were treated with the CTNNB1-CTBP inhibitor ICG-001 (5 mg/kg) or PORCN inhibitor C-59 (20 mg/kg) or saline vehicle by intraperitoneal injection three times per week, from week 21 to 26. At 26 weeks, rats were sacrificed, and uc.158− expression assessed by real-time PCR. Bars represent mean±SD of four samples.

Recent evidence has shown that CCA, another form of primary liver cancer, is dependent on the Wnt pathway despite the lack of *CTNNB1* mutations in malignant cholangiocytes.^27^ ^35–37^ In order to understand if our findings were disease-specific or could be extended also to CCA, we looked at uc.158− expression in an initial cohort of British human intrahepatic CCAs (ICC) and found that uc.158− was significantly overexpressed in tumour tissues compared with healthy livers (figure 5A). Interestingly, uc.158− was increased in CCA compared with normal tissues in all four cases for which matched adjacent tissues were available (figure 5B). These data were confirmed in another series of Italian CCA by in situ hybridisation. We studied the expression of uc.158− using a TMA including 54 ICC, 31 extrahepatic CCA (ECC) and 17 gall bladder cancers (GBC), along with additional 8 normal biliary ducts and liver parenchyma and 6 liver tissues adjacent to CCA (see online supplementary table S6). Normal biliary epithelia and liver parenchyma were always uc.158− negative. High uc.158− expression (++/+++) was detected in 74% of ICC, 58% of ECC and 24% of GBC (figure 5C, see online supplementary table S6). Adequate paired tumoural and adjacent non-tumoural tissues for analysis were available from six cases, and in all of them uc.158− was overexpressed. Interestingly, the perilesional adjacent liver was rarely negative, and most of them showed weak/moderate positivity, suggesting that uc.158− may be involved in the carcinogenic process (see online supplementary figure S5). Stromal cells were strongly positive (see online supplementary figure S4B), in accordance with data by Boulter *et al* 27 showing that the stroma is one of the sources of Wnt in CCA. To further investigate these findings, we studied a rat model of thiocetamide (TAA)-induced CCA, which appropriately models ICC on a background of inflammation and activation of β-catenin, as previously shown.^27^ uc.158− expression was increased in tumour tissues compared with control livers, confirming that uc.158− deregulation is conserved across species along with the sequence (figure 5D). To further prove the dependency on the Wnt pathway, we analysed the expression of uc.158− after treatment with two different Wnt inhibitors. ICG-001 reduced expression of uc.158− by 52%, while C-59 decreased uc.158− expression to almost undetectable levels in rat CCA (figure 5E).

### Biological relevance of uc.158−

**Figure 6:**
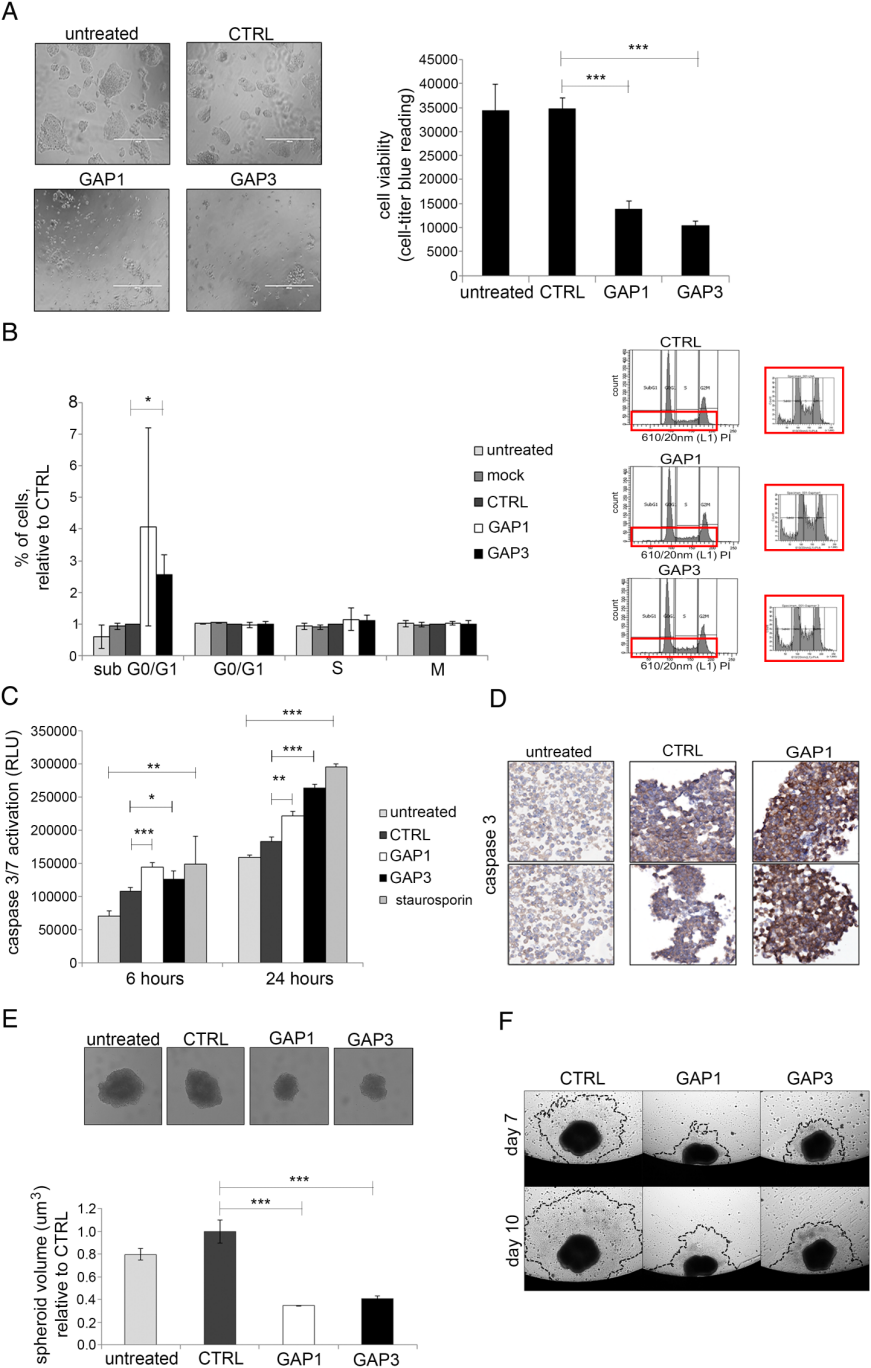
uc.158− inhibition reduces cancer cell growth. (A) HepG2 cells were reverse-transfected with GAPMER probes anti-uc.158− GAP1 and GAP3 or control-scrambled probe (CTRL) in 96 well plates, and cell viability assessed by CellTiter-Blue after 72 hours. Non-transfected (untreated) cells were added as control for transfection toxicity. Representative pictures and quantification of seven replicates with mean±SD are represented. Scale bar: 400 μM. (B) HepG2 cells were transfected, and analysis of cell-cycle distribution was performed by flow cytometry. Quantification of three experiments (mean±SD) is shown on the left. The representative picture on the right shows peaks for cells transfected with CTRL, GAP1 and GAP3 probes. In the red square, representative zoom of the sub-G0/G1 peak. (C) HepG2 cells were reverse-transfected in 96 well plates, and activation of caspase 3/7 was assessed by luminescence assay. Staurosporine was added as positive control. Bars represent mean±SD of three replicates. Asterisks indicate p value by t-test analysis. Analysis of variance comparison was performed comparing all the groups, or only the three experimental groups (CTRL, GAP1, GAP3), with the following results: p:0.0004 and p:0.0012, respectively, at 6 hours; p<0.0001 for both comparisons at 24 hours. (D) HepG2 cells were transfected with GAPMER probe anti-uc.158− GAP1 or CTRL-scrambled probe and then fixed in formalin and embedded in paraffin. Immunohistochemistry was then performed for caspase 3. Representative pictures of two independent experiments are shown. (E) HepG2 cells were reverse-transfected with anti-uc.158− GAP1 and GAP3 or control probe in 100 mm dishes and then plated in ultra low attachment (ULA) 96-well plates to form spheroids. Imaging and quantitation were performed after 7 days with Celigo. Bars represent mean±SD of six replicates. (F) Once the spheroids were formed, they were moved to flat-bottom, gelatin-coated 96-well plates, and migration was assessed at different time points through Celigo.

To gain insights into the biological relevance of uc.158−, we studied phenotypical changes induced by inhibition of uc.158− in HepG2 cells. We tested four different locked nucleic acid probes (GAPMER, GAP1-4) specifically designed to interact with the conserved sequence of uc.158−. GAP1 and GAP3 provided the best inhibition (see online supplementary figure S6) and therefore were selected for further experiments. Cell growth was reduced by 60% and 70% at 72 hours in HepG2 cells transfected with GAP1 and GAP3, respectively, compared with the control (CTRL) probe (figure 6A). To better understand the mechanism through which cell viability is affected, we ran a cell cycle analysis after transfection, and noticed that there were no changes in the progression through the cell cycle phases, but for an increased accumulation of cells in the sub-GO/1 phase (potentially apoptotic) in cells transfected with GAP1 and GAP3 (figure 6B). Indeed, we observed an increase in the activated caspase 3/7 by luminescence (figure 6C) with no increase in necrosis (see online supplementary figure S7). Increase in the expression of caspases and cleaved poly ADP ribose polymerase (PARP) was also noted after uc.158− silencing (figure 6D, see online supplementary figure S8).

Anchorage-independent growth was assessed by tumour 3D spheroid assay. The volume of 3D spheroid generated from HepG2 cells transfected with GAP 1 and GAP3 was reduced to 34% and 40%, respectively, in comparison with spheroids generated from cells transfected with GAP CTRL (figure 6E). In addition, uc.158− silencing reduced tumour spheroid-based migration in HepG2 cells (figure 6F). These data were also confirmed in CCA SW1 cells whose cell viability was dependent on the Wnt pathway (see online supplementary figure S9).

### Uc.158− acts as a competing endogenous RNA

**Figure 7:**
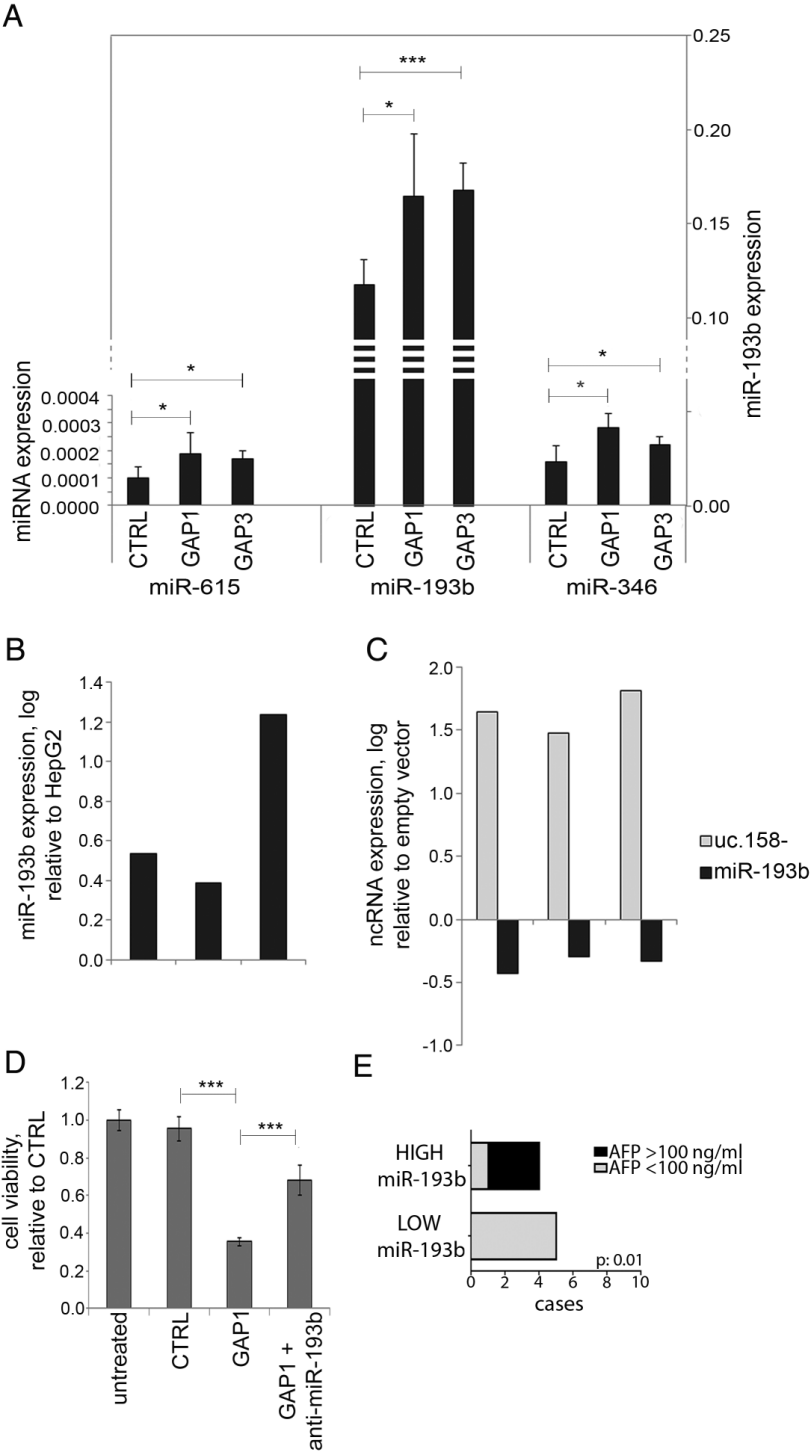
miR-193b expression is dependent on uc.158− expression. HepG2 cells were transfected in 100 mm dishes for 48 hours, and RNA extracted to assess microRNA expression by TaqMan assays. Bars represent mean±SD of three experiments. (B) miR-193b expression was assessed in Huh-7 and HepG2 cells. Bars represent logarithmic expression of miR-193b in Huh-7 versus HepG2. In three independent experiments, miR-193b expression was higher in Huh-7 in comparison with HepG2. (C) Huh-7 cells were transfected with a vector overexpressing uc.158− or an empty vector for 48 hours. Expression of uc.158− and miR-193b was assessed by real-time PCR. Bars represent the logarithmic expression of ncRNAs in cells transfected with uc-158− vector compared with empty vector. The downregulation of mR-193 is quantitatively comparable with the upregulation achieved in panel A. (D) HepG2 cells were transfected with GAPMER control or GAP1 alone or in association with anti-miR-193b, and cell viability assessed by CellTiter-Blue at 48 hours. Bars represent mean±SD of six replicates. (E) Sera were collected from patients with hepatocarcinoma (HCC) naïve for any treatment (n:10), and microRNA extracted using fixed starting volume. miR-193b was assessed by TaqMan assay. χ^2^ test was used to assess the correlation between miR-193b and alpha-fetoprotein (AFP) groups.

Recent evidence suggests that T-UCRs may act as endogenous competing RNAs (ceRNA).^38^ Thus, we searched for potential binding sites for miRNAs in the uc.158− conserved sequence. We shortlisted those miRNAs which are conserved across mouse and human and show a binding site with a predicted ddG score <−10 (see online supplementary figure S10 and table S7). We profiled by NanoString analysis the global expression of miRNAs in the mouse *Apcfl/fl* HCC model. In the hypothesis that uc.158− acts as ceRNA, we searched for miRNAs that were downregulated in *Apcfl/fl* versus *Apc+/+* liver tissues (see online supplementary table S7). Among these nine miRNAs, miR-615, miR-193b and miR-346 were upregulated after inhibition of uc.158− in HepG2 cells, with miR-193b showing the most significant increase and having higher values of expression (figure 7A). Indeed, miR-193b was shown to be downregulated in human HCC.^39^ ^40^ We observed downregulation of miR-193b also in human CCA tissues in comparison with matched adjacent non-neoplastic tissues (see online supplementary figure S11). miR-193b was found to be overexpressed in Huh-7 versus HepG2 cells (in line with uc.158− expression) (figure 7B). Enforced expression of uc.158− in Huh-7 cells induced downregulation of miR-193b (figure 7C). Co-transfection of GAP1 and anti-miR-193b could rescue the biological effect of uc.158− inhibition on cell viability (figure 7D). We then looked at the level of miR-193b in the sera of patients with HCC (n:10) who were naive for any treatment. Patients with low circulating miR-193b had mainly alpha-fetoprotein (AFP) negative tumours, in line with previous reports of observed lower levels of AFP in *CTNNB1-*mutated HCC^41^ (figure 7E).

## Discussion

It is becoming clear that a large number of lncRNAs have a determining role in regulating normal cell physiology as well as a variety of diseases including cancer.^42^ T-UCRs are long ncRNAs that harbour ultraconserved elements and are altered in human carcinomas. Despite some evidence suggests that DNA methylation and miRNA-mediated regulation may be responsible for their modulation,^19^ ^20^ the mechanisms of T-UCR deregulation have been poorly explored. In these studies, we showed that T-UCRs may act downstream of key signalling routes, such as the Wnt pathway, and drive cancer cell growth. Wnt/β-catenin signalling is a key driver in about half of liver cancers, and growing evidence is suggesting that ncRNAs are mediators of this pathway.^17^ ^43^ We provide the first evidence that T-UCRs can be dependent on Wnt activation in a species-conserved fashion. In addition, our studies showed that uc.158− is activated only in cancer cells, suggesting it may be specific for Wnt activation that should be targeted for an antitumoural effect. Indeed, Wnt inhibitor exploitation has been limited by toxicity, and activation of the Wnt pathway in cirrhotic liver may preclude the use of these drugs in patients with liver cancer. Conversely, the identification of a cancer-specific mediator of the pathway may improve the potential of designing novel and more selective drugs. Interestingly, we have shown that molecular traits, such as overexpression of uc.158−, may be present across tumour types independently on site of origin. These findings inform us that T-UCRs may either be used as tissue-specific biomarkers, as in the case of TUC338,^25^ or may be commonly deregulated across tumour types, such as uc.158−. Inclusion criteria with regard to tumour origin (ICC vs ECC) are still a debateable question in the design of clinical trials in CCA. However, based on these studies, we may suggest that clinical trials should be designed on the bases of molecular and genomic features instead of tumour site, and may even include ICC and HCC for selective drugs that target commonly deregulated pathways. As per the reason why uc.158− is aberrantly deregulated in malignant hepatocytes and cholangiocytes, we may speculate that uc.158− overexpressing hepatic cancer stem cells may give rise to specific subtypes of HCC and CCA in the presence of Wnt pathway. However, we cannot exclude that the plasticity of mature hepatocytes to differentiate in cholangiocytes may justify some common traits acquired during liver carcinogenesis.^44^ ^45^ Another potential explanation relies on the evidence that stroma is an important Wnt activator even in tumours, such as CCA, that lack *CTNNB1* mutations.^27^ ^35^ Thus, further studies are warranted to explore the involvement of the stroma-induced activation of uc.158− driven carcinogenesis.

The mechanism of function of T-UCRs is still not clearly elucidated. Previous evidence suggests that they may act as decoy molecules for miRNAs.^38^ We provided data in support of this hypothesis. We have previously shown that miR-193b mediates apoptosis through targeting of mcl-1,^12^ and the overexpression of uc.158− in HCC nicely fits with these data as well as with the previous work that found downregulation of miR-193b in a subgroup of HCCs.^39^ ^40^ It is increasingly recognised that cancer cells rely on a complex network of transcripts generated from coding and non-coding genes, and that interplay of different RNA classes drives cancer progression more than a single aberration.^46^ However, identification of a single molecule representing the activation of a network may prove more useful for the identification of therapeutic targets and for the implementation of screening technologies to be adopted for targeted therapy.
