# Correction: *Wnt signalling modulates transcribed-ultraconserved regions in hepatobiliary cancers*

**DOI:** 10.1136/gutjnl-2016-312278corr1

**Published:** 2024-04-23

Carotenuto P, Fassan M, Pandolfo R, *et al*. Wnt signalling modulates transcribed-ultraconserved regions in hepatobiliary cancers. Gut 2017;66:1268-1277.

The legend of Figure 6D was corrected after publication to clarify the intentional use of reference images of untransfected cells (labelled “untreated”) to show baseline caspase 3 staining in the absence of transfection.

doi:10.1136/gutjnl-2016-312278corr1

