# Supplementary material for: Wnt signalling modulates transcribed-ultraconserved regions in hepatobiliary cancers
Source: Gut. 2016 Sep 12;66(7):1268–77. doi: 10.1136/gutjnl-2016-312278 (PMC5530482; doi:10.1136/gutjnl-2016-312278)

SUPPLEMENTARY METHODS

Cell lines. The human cell lines HepG2, PLC/PRF-5, Huh-7, Hep-3B were obtained from the American Type Culture Collection (ATCC) (Manassas,VA, USA). Normal human hepatocytes were obtained from Sciencell (Carlsbad, CA, USA). SW1 malignant cholangiocytes were kindly provided by Stuart J Forbes (University of Edinburgh). All cancer cells were cultured in Dulbecco's Modified Eagle Media with 10% fetal bovine serum (FBS), at 37°C with 5% carbon dioxide. Normal human hepatocytes were culture in Hepatocyte Medium (Sciencell) with 5% FBS, and 1% hepatocyte growth serum (Sciencell). All cells were regularly subjected to Mycoplasma testing.

RNA extraction. RNA was extracted by using Trizol reagent and following manufacturer's instruction (Invitrogen, Carlsbad, CA, USA). For RNA extraction from FFPE human tissues, tumour and adjacent components were microdissected and processed with RecoverAll Total Nucleic Acid Isolation Kit following manufacturers' instructions (Life Technologies, Paisley, UK).

T-UCR expression profiling in human cell lines. Data for uc.158- expression were retrieved from a microarray LncRNA profiling performed with the human LncRNA Array v2.0 (Arraystart) and already reported in Braconi *et al*.[1] For this specific profiling one replicate for each cell line was available. The array included also >20000 mRNA transcripts. Base on the KEGG (Kyoto Encyclopedia of Genes and Genomes, http://www.genome.jp/kegg) database, pathway analysis for differentially expressed mRNAs was derived. This analysis allowed determining the biological pathway for which there was a significant enrichment of differentially expressed mRNAs. The p-value denotes the significance of the Pathway. We looked at the Wnt pathway and reported p values for specified comparison in Supplementary Table 4.

Real-time PCR analysis. One μg total RNA was reverse transcribed to cDNA and quantitative real time PCR performed using specific primers (Suppl Table 1). Expression was

normalized to that of GAPDH. Genomic contamination was excluded by PCR of controls lacking reverse transcriptase for each sample. miRNA expression was assessed by Taqman Assay and normalized to that of RNU48 (Life Technologies, Paisley, UK)

NanoString. miRNA expression profiling was analysed with nCounter from NanoString Technologies (Seattle, WA, USA), using the nCounter Mouse miRNA Expression assay kit, as previously described.[2] Technical normalization was performed using the synthetic positive controls to adjust the counts for each miRNA target in that assay. Then biological normalization was performed to correct for differences in sample abundances. Each sample was normalized to the geometric mean of the top 50 most highly expressed miRNAs. Student's t test was used on normalized counts to calculate statistical significances of pairwise comparisons.

Rapid Amplification of cDNA ends (RACE). RNA from the liver of *Apcfl/fl* mouse was used to generate RACE-ready cDNA using the SMARTer RACE cDNA amplification Kit (Clontech, Mountain View, CA, USA) following the manufacturer's protocol. Amplification of cDNA ends was performed by using Universal Primer mix (UPM) and gene-specific primers. Heart mouse RNA and Transferrin-Receptor-specific primers provided by the manufacturer were used as controls. In case the primary PCR reaction failed to give distinct bands, we performed a "nested" PCR using the nested universal primer. PCR products were separated in a 1.5% agarose gel, and DNA extracted, cloned in the TOPO.TA.2 plasmid and sequenced using the 48-capillary Applied Biosystems 3730 DNA Analyzer (Applied Biosystem, Foster City, CA, USA).

Transfection. Cells were transfected using HiPerfect (Qiagen, Hilden, Germany) according to the manufacturer's protocol. siRNA against *CTNNB1* (Thermo-scientific, Hemel, UK) was used at 50 nM for 72 hours. For uc.158- silencing LNA probes control, and LNA anti-uc.158- (GAPMERs, Exiqon, Vedbaek, Denmark) were used at a final concentration of 25nM.

Western Blotting. Cells were collected and protein extracted. Immunoblot analysis was performed as previously described.[3] The primary antibodies used were as follows: mouse monoclonal actin (MP Biomedical, Santa Ana, CA, USA), rabbit polyconal PARP (9542 Cell Signalling, Danvers, MA, USA), rabbit monoclonal cleaved-PARP (D64E10 Cell Signalling), and mouse monoclonal caspase 9 and cleaved caspase 3 (7237 and 9664 Cell Signalling), and mouse monoclonal β-catenin (D-10, Santa Cruz Biotechnology, Santa Cruz, CA, USA).The latter recognizes the C-terminus of β-catenin and therefore identifies two bands in HepG2 cells that harbour also a truncated form of β-catenin as result of their mutation.

Immunohistochemistry: Immunohistochemical analysis for human caspase 3 (clone E-8; S Santa Cruz Biotechnology, Santa Cruz, CA, USA), human β-catenin (760-4242, Ventana Medical Systems, Milan, Italy) was performed on the Benchmark LT automated system from Leica Microsystems Bondmax (Leica, Wetzlar, Germany) according to the manufacturer's specifications.

Immunofluorescence Microscopy. Cell monolayers were fixed in 4% formaldehyde, washed with PBS three times and blocked in 5% BSA for 1 hour at room temperature, then incubated with mouse monoclonal β-catenin antibody (1:100; Santa Cruz Biotechnology). After 1 hour incubation, cells were washed serially with PBS and incubated for another 1 hour with fluorochrome-conjugated secondary antibodies at room temperature. After washing with PBS, cells were incubated for 10 minutes with nuclear dye (Hoechst 1ug/ml; Pierce, Rockford, IL, USA). Cells were then mounted in a coverslip glass and fluorescence visualized and captured using a Carl Zeiss confocal imaging system (Carl Zeiss, Heidenheim, Germany).

***In Situ* RNA hybridization.** A locked nucleic acid (LNA) probe with complementarity to a 21-bp section of uc.158- was labeled with 5′-digoxigenin and synthesized by Exiqon. Tissue sections on the tissue microarray were digested with ISH protease 1 (Ventana Medical Systems) and *in situ* hybridization performed as described.[3] Negative controls included

omission of the probe and the use of a scrambled LNA probe. Each sample was classified by two independent reviewers according to a 4-tiered scoring system based on the intensity of uc.158- expression as follows: 0: indicates no stain or stain in less than 10% of tumor cells; 1+: faint/weak cytoplasm/nuclear stain in 10% or more of cells; 2+: moderate cytoplasm/nuclear stain in 10% or more of tumor cells; and 3+: strong cytoplasm/nuclear stain in 10% or more of tumor cells. In all the considered tissue samples, fibroblasts, lymphocytes, and endothelia featured uc.158- cytoplasm/nuclear expression and were assumed as positive internal control (not considered in ISH score).

Anchorage-dependent cell growth. Cells were reverse- transfected with HiPerfect (Qiagen) in 96 well plates and cell viability assessed by fluorescent method (CellTiter-Blue® Cell Viability Assay, Promega, Madison, WI, USA).

Cell cycle analysis. Cells were transfected with LNA probes using HiPerfect and plated in 10%FBS medium. After 24 hours cells were starved for 24 hours and then 10%FBS serum was added to the medium. After further 24 hours cells were stained with Propridium iodide and analysed with BD LSRII 13 parameter analyser and BD FACSDiVa software (BD Bioscience, Oxford, UK).

3D-Spheroid formation. Cells were subjected to reverse transfection using Hiperfect in 100 mm dishes as per the manufacturer’s instruction. After 24 hours cells were plated in UltraLow Attachment (ULA) round bottom 96 well plates (Corning, Corning, NY, USA) and 3D-spheroid formation analysed with Celigo S (Nexcelom, Manchester, UK) as previously described.[4] After 7 days, spheroids were transferred to a flat bottom plate previously coated with 0.1% gelatin and cell migration analysed as previously described.[5]

Caspase 3/7 activation. Cytotoxicity and caspase 3/7 activation were measured by using the ApoTox-Glo™ Triplex Assay and following manufacturers’ instruction.

Cloning uc.158-. The ultraconserved region uc.158- was cloned in the pBABE-puro plasmid (Addgene, Cambridge, MA, USA). Genomic DNA from HepG2 was used as source DNA.

The upstream primer contained the *BamHI* restriction site, while the downstream primer contained the *SalI* restriction site. An empty vector was used as a control.

Circulating miRNA. HCC patients' serum was collected and stored at -80. RNA was extracted from 200uL using the miRCURY RNA isolation kit (Exiqon) following supplier instructions. Fixed starting volume was maintained for each sample. 2ul of RNA was then reverse transcribed and assessed for miR-193b expression (Taqman, Lifetechnologies, Paisley UK).

Compounds: ICG-001 and Staurosporine was purchased from Tocris Bioscence (Bristol, UK), C-59 from Cellagen Technologies (San Diego, CA, USA).

SUPPLEMENTARY TABLES

Supplementary Table 1. List of primers

Primer	Sequence
**human GAPDH F**	CGACAGTCAGCCGCATCTT
**human GAPDH R**	CCCCATGGTGTCTGAGCG
**rat GAPDH F**	GGGTGTGAACCACGAGAAT
**rat GAPDH R**	ACTGTGGTCATGAGCCTTC
**mouse GAPDH-F**	AATGTGTCCGTCGTGGATCTGA
**mouse GAPDH-R**	GATGCCTGCTTCACCACCTTCT
**UC.158- F2**	CATCCAGTTTGCGCTAATCA
**UC.158- R2**	TGAACGAGCTGCTGGTAGAC
**UC.455+ F1**	GGACCACGGTACAGCGATAA
**UC.455+ R1**	CTTCAGCGTGATGAATAAATGC
**UC.196+ F1**	TCCAAATCTCACTCAAGTGGAAC
**UC.196+ R1**	CGTGAGCTCCTTGACACAAA
**UC.82- F1**	GTGTTTAAATCAATCAACTGCAAAC
**UC.82- R1**	TTCAGTGTGGGATCACAGTAGG
**human survivin F**	CCAGATGACGACCCATAGA
**human survivin R**	TCCGCAGTTTCCCTCAAATTC
**human cyclin D1 F**	TCC TCT CCA AAA TGC CAG AG
**human cyclin D1 R**	TGA GGC GGT AGT AGG ACA GG
**human CTNNB1F**	TGTTCTTGAGTGAAGGACTGA
**human CTNBB1 R**	AAAATCCAGCGTGGACAATGG
**ARLong for RACE uc.158-**	TGGTAGACGGGAACAGGGCCGCTGCTGGA
**uc158- cloning primer F**	GCggatccAGCCTGCACGTTTCTCTTTG
**uc158- cloning primer R**	GCgtcgacCCGCTCGGCCTTAATTGATA

Supplementary Table 2. List of T-UCRs which are aberrantly expressed in *Apcfl/fl* HCC compared to *Apc+/+* normal liver.

T-UCR	***Fold Change***	***p-value***	***Location***	***Relationship to other genes***	***associated gene***
uc.97+	3.79	0.0138	chr2	sense overlap	Hat1
uc.158-	3.73	0.000005	chr13	intergenic	
uc.163-	2.40	0.0041	chr13	intergenic	
uc.285+	2.12	0.0054	chr10	sense overlap	Ccar1
uc.30+	-2.01	0.0002	chr3	intergenic	
uc.258+	-2.04	0.0089	chr4	sense overlap	Zcchc7
uc.186-	-2.20	0.0321	chr11	sense overlap	Hnrnph1
uc.414-	-2.24	0.0111	chr11	sense overlap	Nr1d1
uc.196+	-2.27	0.0493	chr4	intergenic	
uc.18-	-2.60	0.0001	chr4	sense overlap	Eri3
uc.460-	-2.65	0.0004	chrX	antisense overlap	Pola1
uc.82-	-2.68	0.0048	chr2	intergenic	
uc.193-	-2.68	0.0047	chr9	sense overlap	Syncrip
uc.50-	-2.89	0.0382	chr17	sense overlap	Srsf7
uc.371-	-2.96	0.0001	chr12	sense overlap	Ralgapa1
uc.1+	-3.05	0.0024	chr4	sense overlap	Pex14
uc.418-	-3.17	0.0181	chr11	sense overlap	Srsf1
uc.77+	-3.21	0.0230	chr2	antisense overlap	Zeb2
uc.153+	-3.33	0.0008	chr13	sense overlap	Tnpo1
uc.455-	-5.33	0.0091	chr2	sense overlap	Rbm39
uc.455+	-5.43	0.0281	chr2	antisense overlap	Rbm39
uc.64+	-7.19	0.0022	chr11	sense overlap	Ehbp1

Supplementary Table 3. *Apcfl/fl* HCC, *Apc*+/+ normal liver and DEN-HCC tissues have been profiled for T-UCR expression. Listed are the T-UCRs which can differentiate *Apcfl/fl* HCC from *Apc*+/+ normal liver as well as *Apcfl/fl* HCC from DEN-induced HCC.

T-UCR	***APC fl/fl vs APC +/+***	***APC fl/fl vs DEN***	***Location***	***Relationship to other genes***	***associated gene***
uc.158-	3.73	2.21	chr13	intergenic	
uc.196+	-2.27	-6.40	chr4	intergenic	
uc.82-	-2.68	-2.62	chr2	intergenic	
uc.455+	-5.43	-3.65	chr2	antisense overlap	*RBM*39

Supplementary Table 4. Global gene expression profiling has been performed with human LncRNA Array v2.0 (Arraystar) and already reported in [1]. For this specific profiling one replicate for each cell line was available. The array included also >20000 mRNA transcripts. Base on the KEGG (Kyoto Encyclopedia of Genes and Genomes, http://www.genome.jp/kegg) database, pathway analysis for differentially expressed mRNAs was derived. This analysis allowed determining the biological pathway for which there was a significant enrichment of differentially expressed mRNAs. The p-value denotes the significance of the Pathway. We looked at the Wnt pathway and reported p values for specified comparison. HH: Normal human hepatocytes.

Pathway	Sample comparison	P value
Wnt signalling	HepG2 vs HH	0.04
Wnt signalling	Huh-7 vs HH	Not found

Supplementary Table 5. Human FFPE liver tissues (2 cirrhosis, 8 HCC, 1 Hepatoblastoma) were stained for b-catenin by IHC and for uc.158- by ISH.

			**uc.158-,** -/+	**uc.158-,** ++/+++
**b-catenin**	**tumour**	negative	1	0
		membranous or cytoplasmic	2	1
		nuclear	1	4
	**non tumour**	membranous or cytoplasmic	1	0
		nuclear	1	0

Supplementary Table 6. A tissue microarray of human liver tissues has been assessed for uc.158- expression by ISH. Three tissue cores were analysed per each case. Each sample was classified by two independent reviewers according to a 4-tiered scoring system (as per methods).

Lesions	uc.158- scale				uc.158- dichotomized	
	-	+	++	+++	-/+	++/+++
Normal biliary epithelium (n=8)	8	0	0	0	8	0
%	100	0	0	0	100	0
Normal liver parenchyma (n=8)	8	0	0	0	100	0
%	100	0	0	0	100	0
Adjacent liver parenchyma (n=6)	1	4	1	0	5	1
%	16.7	66.7	16.7	0	83.3	16.7
ICC (n=54)	3	11	24	16	14	40
%	5.6	20.4	44.4	29.6	25.9	74.1
ECC (n=31)	3	10	6	12	13	18
%	9.7	32.3	19.4	38.7	41.9	58.1
Gallbladder cancer (n=17)	5	8	4	0	13	4
%	29.4	47.1	23.5	0	76.5	23.5

Supplementary Table 7. microRNA profiling was performed by NanoString in the liver of *Apcfl/fl* and *Apc+/+* mice. Below are the data of expression for the microRNAs that are predicted to have binding sites in the uc.158- sequence with ddG score <-10 and are conserved across mouse and human. In red are highlighted those that were down-regulated in *Apcfl/fl* in comparison to *Apc+/+*.

microRNA	Number of Sites	ddG Score	APC fl/fl vs APC+/+, log
miR-339-5p	2	-22.46	-0.11
miR-219a-2-3p	1	-13.95	-0.60
miR-187	3	-14.03	-1.04
miR-615-5p	1	-13.34	-0.33
miR-346	2	-13.19	-0.66
miR-127-5p	2	-12.04	4.71
miR-668	3	-11.95	0.23
miR-16	5	-11.17	0.20
miR-874	3	-10.59	-0.61
miR-128	1	-10.55	0.16
miR-193b	2	-10.27	-0.33
miR-758	3	-10.16	-0.16
miR-139-3p	1	-10.03	-0.74

SUPPLEMENTARY FIGURE LEGEND

Supplementary Figure 1. Mice were treated with i.p. DEN and sacrificed at 36 weeks. *(A)* Number of tumours in the liver of animals treated with vehicle or DEN. *(B)* Immunohistochemistry for β-catenin in HCC tissues from mice treated with DEN. They do not show nuclear localization of β-catenin as opposite to the tumours in *Apcfl/fl* mice (as previously shown).

Supplementary Figure 2. (*A*) Western blotting was performed for proteins responsive to activation of the Wnt pathway in cell lysates from Huh-7 and HepG2 cells. (*B*) Expression data for genes that are responsive to Wnt activation has been retrieved by the global gene expression profiling performed with human LncRNA Array v2.0 (Arraystar) already reported in [1]. Bars represent log value of expression in HepG2 relative to Huh7. Genes responsive to Wnt pathway are mainly expressed in HepG2. *(C)* Data for uc.158- were retrieved from a T-UCR expression profile performed in normal human hepatocytes (HH) and malignant hepatocytes previously reported in [1]. Bars represent uc.158- expression relative to HH. In this profiling only one replicate per sample was available.

Supplementary Figure 3. (*A*) Schematic representation of the uc.158- region and the probes used for uc.158- characterization. (*B*) RNA from *Apcfl/fl* mouse was used to perform 5-RACE. TRF probes were used as positive control. Results for the first and the nested run are shown. The band was cut from the gel and sequenced. (C) Human liver tissues were used to test LNA-anti-uc.158- probes for ISH. Bars indicate 300um. (D) ISH for uc.158- in human liver tissues. Necrotic areas were negative for uc.158-, confirming the specificity of the probe. Bar 200um.

Supplementary Figure 4. (*A*) ISH of human liver tissue with hepatitis. Inflammatory lymphocytes are positive for uc.158-. (*B*) ISH of human CCA tissue. Peritumoural stroma is strongly positive for uc.158-. Bar 400um.

Supplementary Figure 5. ISH of human liver tissues. Non-cancerous liver tissue adjacent to CCA tissue is mildly positive for uc.158-, while liver from healthy control is negative. Bar 200um.

Supplementary Figure 6. (**A**) HepG2 cells were transfected with GAPMER probes anti-uc.158- GAP1-4 for 48 hours, then fixed in formalin and embedded in paraffin. ISH showed inhibition of uc.158- with GAP1 and GAP3. (*B*) HepG2 cells were transfected with LNA GAP CTRL, GAP1 and GAP3 for 48 hours and uc.158- expression was assessed by real time PCR.

Supplementary Figure 7. HepG2 cells were reverse-transfected in 96 well plates and cytotoxicity was assessed by fluorimetric assay. Staurosporine was added as positive control. Bars represent mean+/-SD of 3 replicates. Transfection was toxic, but there was no increase in cytotoxicity (necrosis) from the inhibition of uc.158- compared to LNA GAP CTRL.

Supplementary Figure 8. HepG2 cells were transfected for 24 hours and lysates obtained. Western blotting for the indicated antibodies was performed.

Supplementary Figure 9. (*A*) SW1 cells were selected amongst a panel of human CCA cell lines because dependent on Wnt. Indeed treatment with different Wnt-inhibitors caused the greatest dose-dependent effect in SW1 cells. (*B*) SW1 cells were reverse-transfected with GAPMER probes anti-uc.158- GAP1 or control scrambled probe (CTRL) in 96 well plates and cell viability assessed by Cell Titer blue. *(C)* SW1 were reverse-transfected with anti-uc.158- GAP1 or control probe in 100 mm dishes and then plated in ULA 96-well plates to form spheroids. Imaging and quantitation were performed after 4 days with Celigo. Bars represent mean +/-SD of 4 replicates. (*D*) Western blotting imaging of SW1 cells treaed with GAP1 or GAP-CTRL and assessed for cleaved caspase 3. Cells treated with DMSO or staurosporin (from a different experiment) were added as positive control.

Supplementary Figure 10. Circos plots showing the potential binding sites for all miRNAs (left) or those miRNA with ddG score <10 (right) within the uc.158- ultraconserved region.

Supplementary Figure 11. Human CCA FFPE tissues were microdissected to obtain tumour and adjacent non neoplastic compartments. miR-193b expression was assessed in each component for each patient by using Taqman assays, and presented as the ratio between tumour and adjacent in Log scale. Bars represent mean of two technical replicates for each patient. Paired two-tailed ttest analysis between miR-193b expression in tumour and adjacent tissues confirmed a statistically significant difference (p:0.001).

SUPPLEMENTARY REFERENCES

1. Braconi C, Kogure T, Valeri N, et al. microRNA-29 can regulate expression of the long non-coding RNA gene MEG3 in hepatocellular cancer. *Oncogene* 2011;30(47):4750-6.
2. Valeri N, Braconi C, Gasparini P, et al. MicroRNA-135b promotes cancer progression by acting as a downstream effector of oncogenic pathways in colon cancer. *Cancer Cell* 2014;25(4):469-83.
3. Braconi C, Valeri N, Kogure T, et al. Expression and functional role of a transcribed noncoding RNA with an ultraconserved element in hepatocellular carcinoma. *Proc Natl Acad Sci U S A* 2011;108(2):786-91.
4. Vinci M, Gowan S, Boxall F, et al. Advances in establishment and analysis of three-dimensional tumor spheroid-based functional assays for target validation and drug evaluation. BMC Biol. 2012 Mar 22;10:29.
5. Vinci M, Box C, Zimmermann M, et al. Tumor spheroid-based migration assays for evaluation of therapeutic agents. Methods Mol Biol. 2013;986:253-66.

Supplementary figures

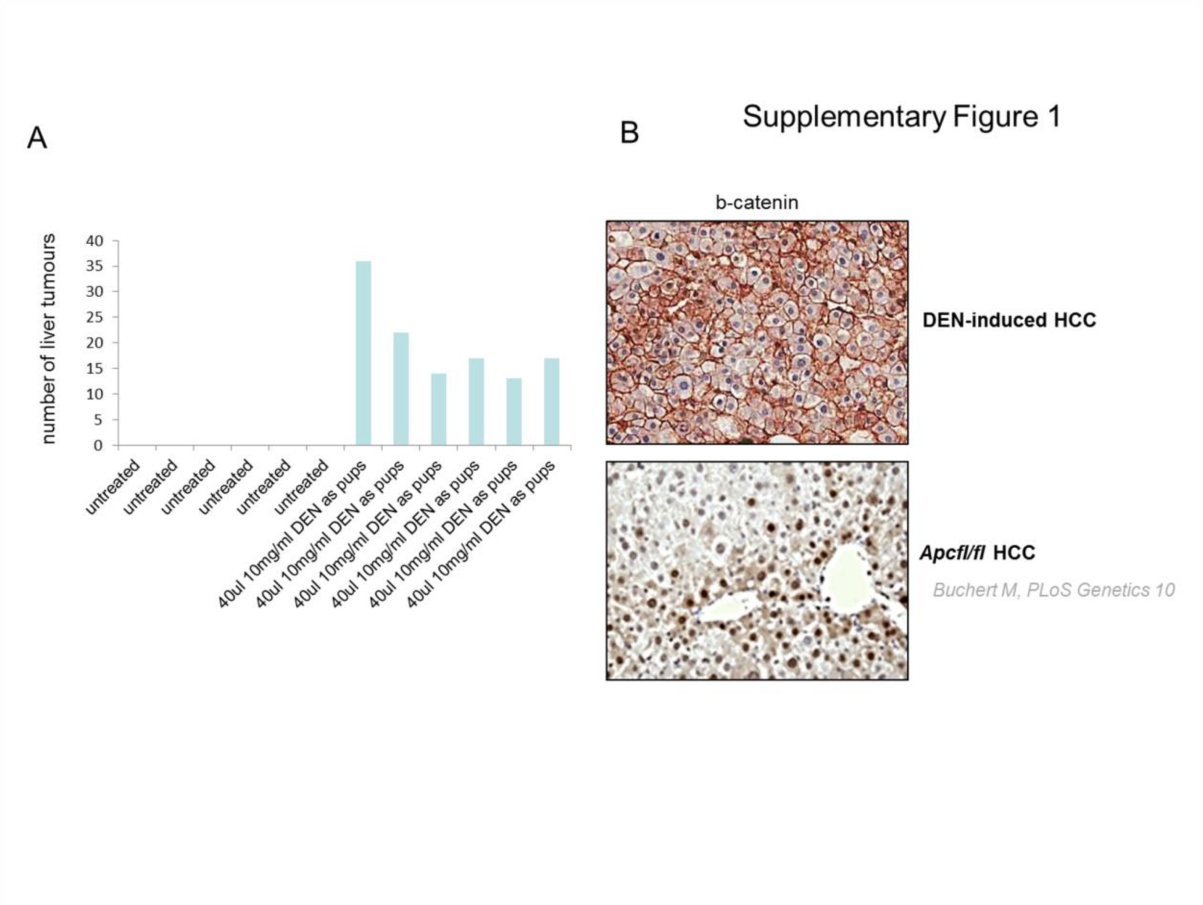

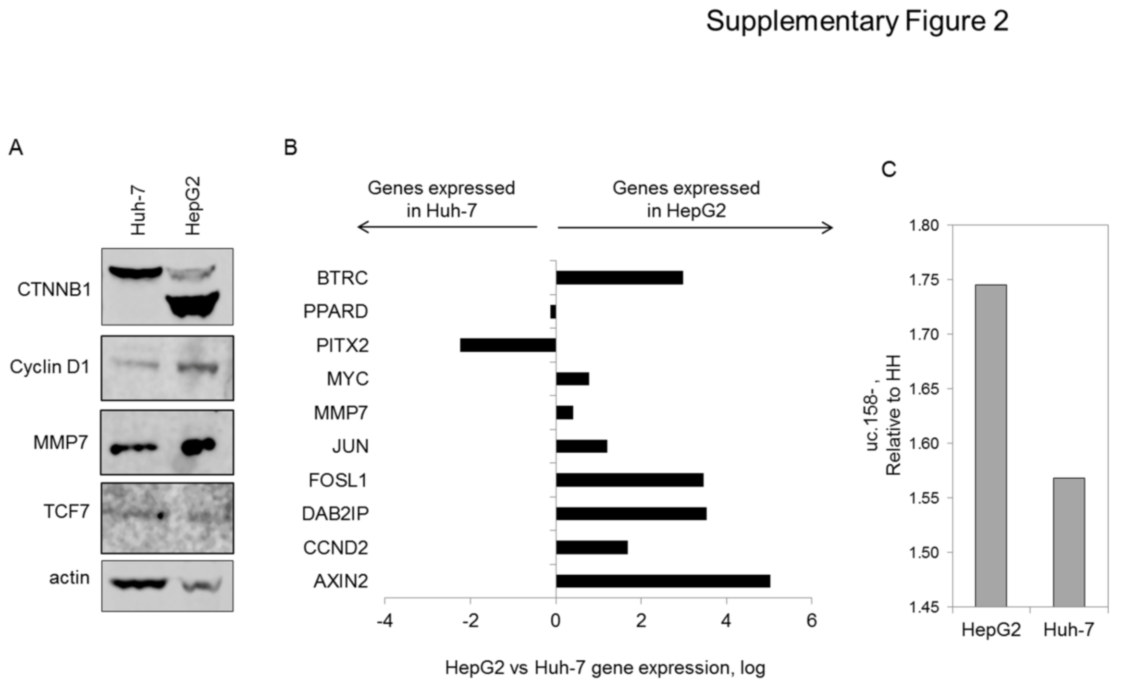

Supplementary Figure 3

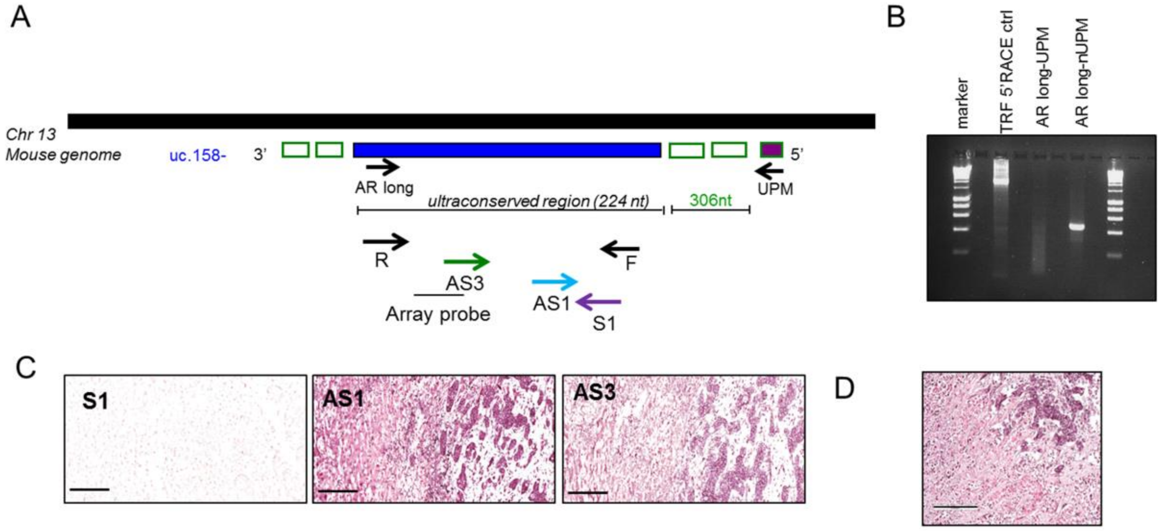

Supplementary Figure 4

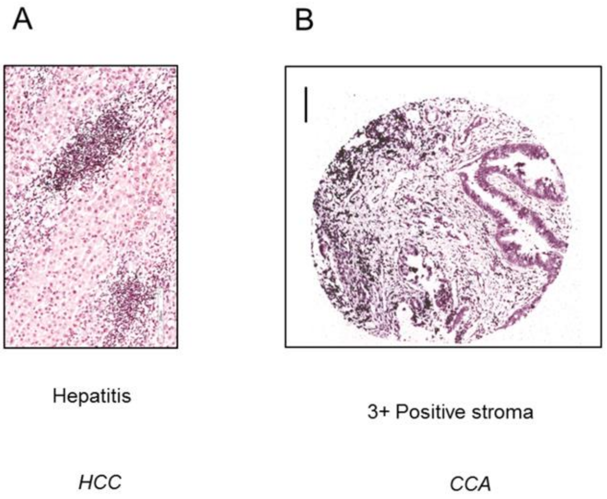

Supplementary Figure 5

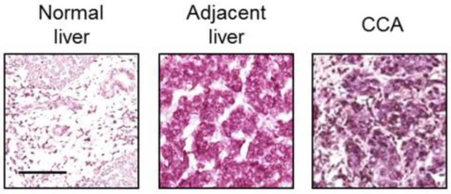

Supplementary Figure 6

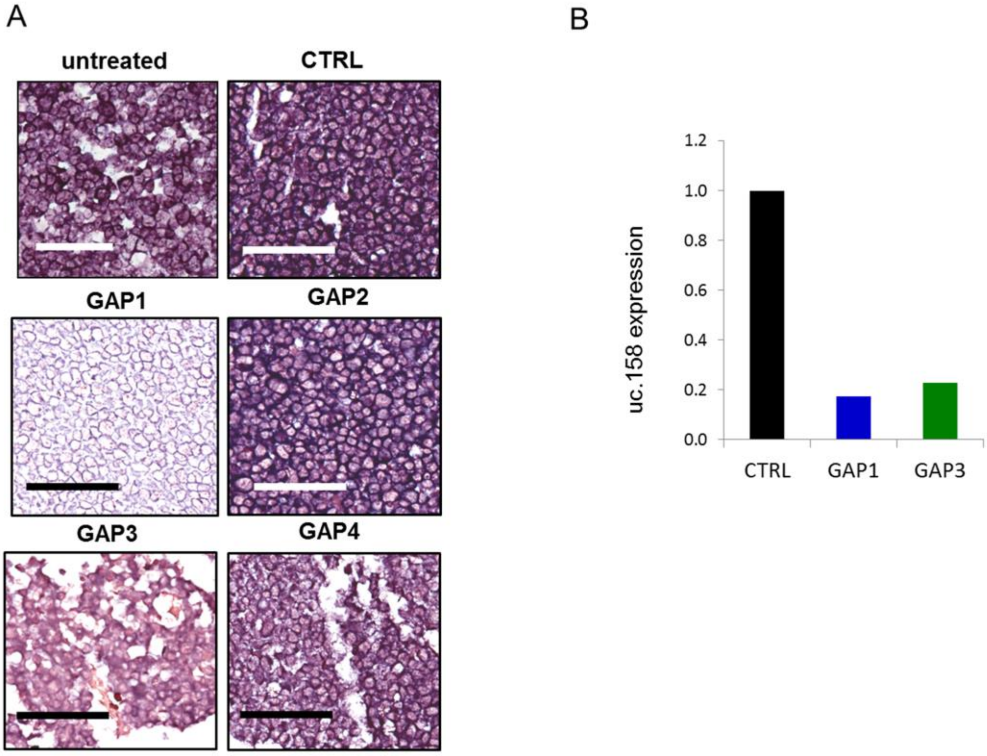

Supplementary Figure 7

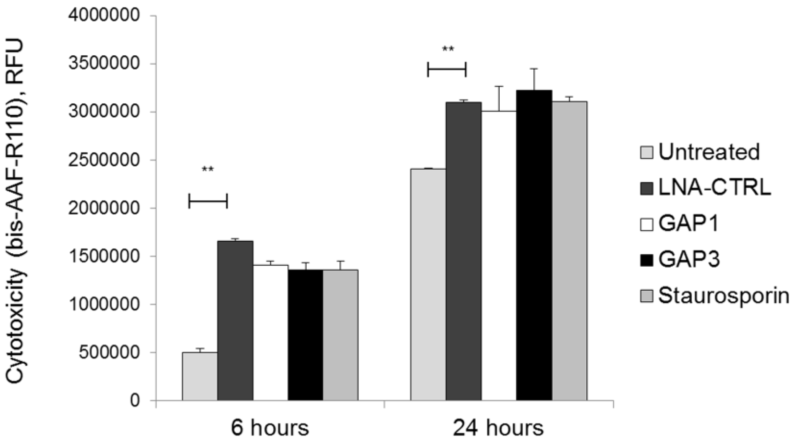

Supplementary Figure 8

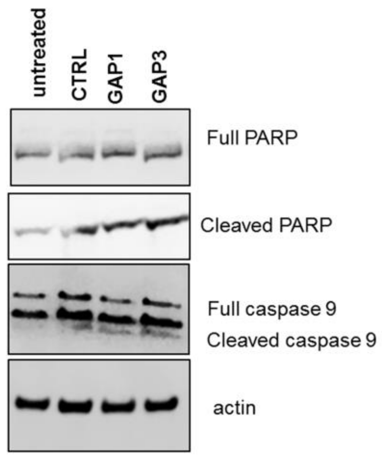

Supplementary Figure 9

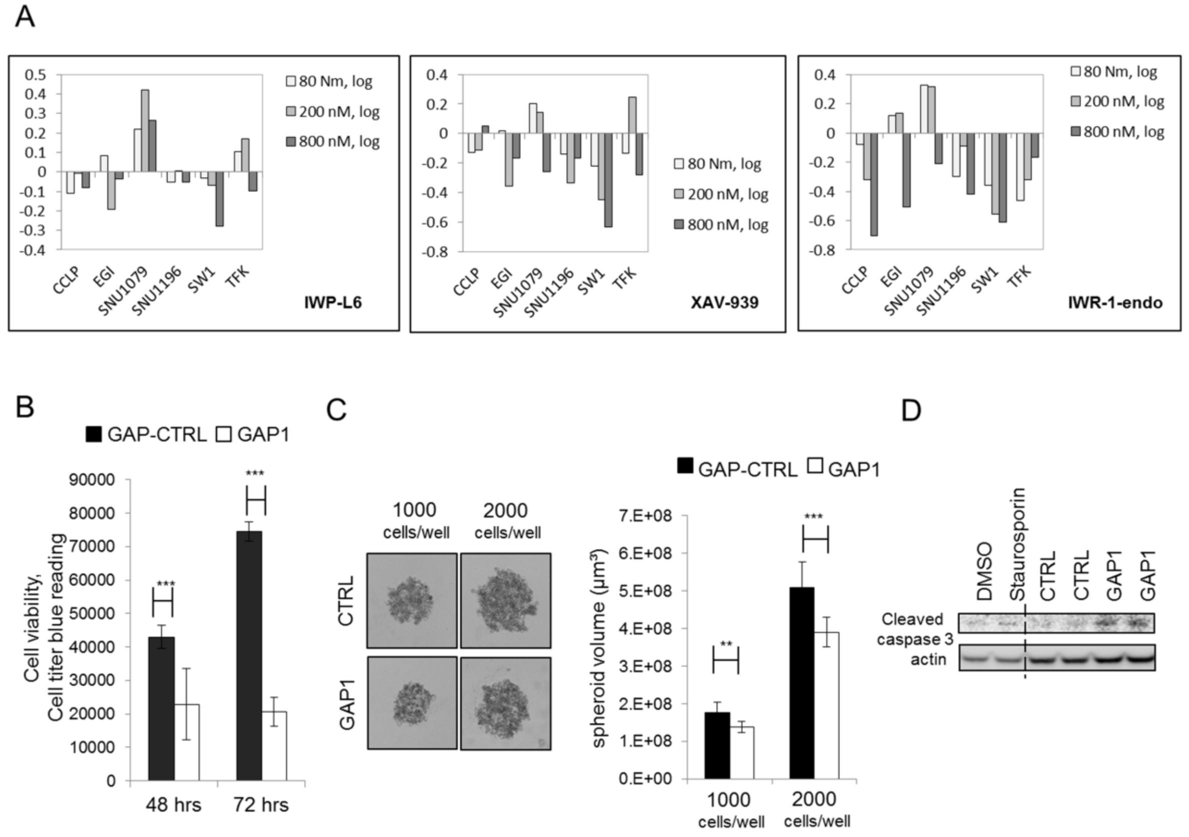

Supplementary Figure 10

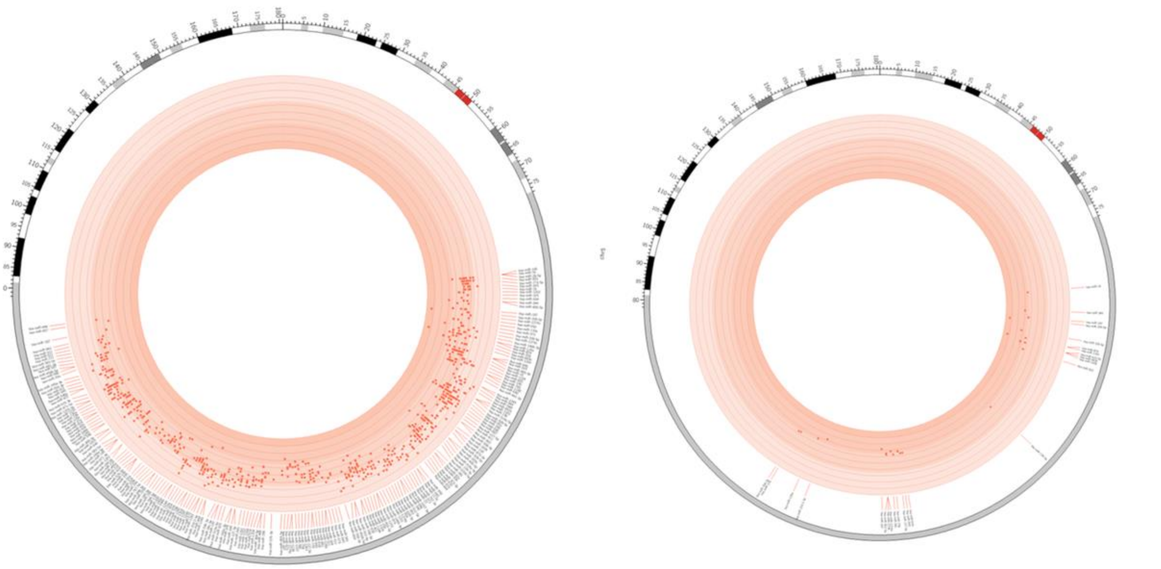

Supplementary Figure 11

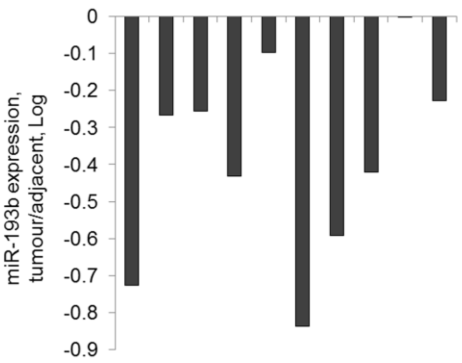

Supplement: supplementary data [file gutjnl-2016-312278supp001.pdf]
